# Neural effects of antidepressant medication and psychological treatments: a quantitative synthesis across three meta-analyses

**DOI:** 10.1192/bjp.2021.16

**Published:** 2021-10

**Authors:** Camilla L. Nord, Lisa Feldman Barrett, Kristen A. Lindquist, Yina Ma, Lindsey Marwood, Ajay B. Satpute, Tim Dalgleish

**Affiliations:** 1 Medical Research Council Cognition and Brain Sciences Unit, University of Cambridge, UK; 2 Department of Psychology, Northeastern University, Boston, Massachusetts, USA; 3 Department of Psychology and Neuroscience, University of North Carolina, Chapel Hill, North Carolina, USA; 4 State Key Laboratory of Cognitive Neuroscience and Learning, IDG/McGovern Institute for Brain Research, Beijing Normal University, China; and Chinese Institute for Brain Research, Beijing, China; 5 Department of Psychological Medicine, Institute of Psychiatry, Psychology & Neuroscience, King's College London, UK

**Keywords:** Antidepressants, cognitive–behavioural therapies, anxiety disorders, depressive disorders, imaging

## Abstract

**Background:**

Influential theories predict that antidepressant medication and psychological therapies evoke distinct neural changes.

**Aims:**

To test the convergence and divergence of antidepressant- and psychotherapy-evoked neural changes, and their overlap with the brain's affect network.

**Method:**

We employed a quantitative synthesis of three meta-analyses (*n* = 4206). First, we assessed the common and distinct neural changes evoked by antidepressant medication and psychotherapy, by contrasting two comparable meta-analyses reporting the neural effects of these treatments. Both meta-analyses included patients with affective disorders, including major depressive disorder, generalised anxiety disorder and panic disorder. The majority were assessed using negative-valence tasks during neuroimaging. Next, we assessed whether the neural changes evoked by antidepressants and psychotherapy overlapped with the brain's affect network, using data from a third meta-analysis of affect-based neural activation.

**Results:**

Neural changes from psychotherapy and antidepressant medication did not significantly converge on any region. Antidepressants evoked neural changes in the amygdala, whereas psychotherapy evoked anatomically distinct changes in the medial prefrontal cortex. Both psychotherapy- and antidepressant-related changes separately converged on regions of the affect network.

**Conclusions:**

This supports the notion of treatment-specific brain effects of antidepressants and psychotherapy. Both treatments induce changes in the affect network, but our results suggest that their effects on affect processing occur via distinct proximal neurocognitive mechanisms of action.

Thousands of controlled trials support the efficacy of psychological therapy and antidepressant medication to treat emotional disorders. Combining psychotherapy and antidepressants enhances therapeutic response, suggesting complementary proximal mechanisms.^1^ Influential neural theories conceptualise psychotherapy as targeting affect circuitry via prefrontal cortical mechanisms, and antidepressants as altering affect processing directly via effects on subcortical structures such as the amygdala.^1^

There is substantial evidence that both psychotherapy and antidepressant medication alter emotion and reward processing, thereby normalising affective processing.^2,3^ However, they are thought to change affective processing via distinct (different) cognitive routes. For example, psychotherapy may change cognitive control of affect processing^2^ or attention and awareness of affective state,^4^ whereas antidepressants may alter generation of affective and visceral sensations.^2,5^ Different proximal mechanisms of psychotherapy and antidepressants might explain the differing outcomes of these treatments, including the particular advantage of psychotherapy compared with antidepressants in relapse prevention.^1,6^ Evidence for distinct cognitive mechanisms would be supported by distinct neural changes following antidepressant medication and psychotherapy; evidence against this theory would be supported if only overlapping changes were evoked by the two treatments.

Theories of different proximal mechanisms have now been tested using neuroimaging (such as functional magnetic resonance imaging) to measure brain activation before and after a typical course of antidepressants or psychotherapy. Some empirical work is supportive of differential mechanisms. A number of studies show changes in activation in the amygdala, hippocampus or other subcortical regions as a result of antidepressant medication (e.g.^7^), whereas changes in regions of the prefrontal cortex are commonly reported following psychotherapy (e.g.^8,9^). However, when directly contrasting the two, distinct mechanisms are not always found. A recent trial randomised 55 people with anxiety or depression to 12 weeks of a selective serotonin reuptake inhibitor (SSRI) or cognitive–behavioural therapy (CBT), measuring brain activation during affect processing before and after treatment.^10^ This study found only treatment-general effects on the brain: overlapping neural changes in the limbic system following CBT and SSRIs.^10^ Discrepancies between studies may occur because individual trials with neuroimaging measures represent sample- or intervention-specific findings, and suffer from relatively low statistical power.

Neuroimaging meta-analysis is a statistically powerful and generalisable approach to elucidate whether neural changes from psychotherapy and antidepressant medication reliably diverge or converge. Neuroimaging meta-analysis performs stringent statistical testing of the activation patterns obtained from many studies to determine whether activation occurs within particular brain regions across studies by chance (i.e. is not statistically significant) or if it reliably converges in the same brain regions across studies (i.e. is statistically significant). Recently, two meta-analyses separately reported the effects of antidepressants and psychotherapy on neural activation.^11,12^ However, a quantitative comparison of such effects is critical to examining neural divergence of treatment approaches.

Using primary data from these two meta-analyses, we tested whether treatment with antidepressants or psychotherapy evoked overlapping and/or distinct neural changes. We then separately tested whether neural changes from antidepressant medication or psychotherapy overlapped with known affect circuitry, using data from a third meta-analysis of affective processing in the brain.^5^ This allowed us to directly test whether or not the proximal mechanisms of treatment were overlapping or distinct (or both) for antidepressants and psychotherapy. In line with influential theoretical models, we anticipated that both psychotherapy and antidepressants would evoke changes in the affect network, but that psychotherapy would change prefrontal regions involved in attention and awareness of affect processing whereas antidepressants would change subcortical regions involved in the generation of affective and visceral sensations.

## Method

We employed activation likelihood estimation (ALE), one of the more commonly used algorithms for coordinate-based meta-analysis, to test for convergence and divergence of antidepressant and psychotherapy effects, and their overlap with the affect network. In ALE analysis, coordinates from each neuroimaging study are treated as three-dimensional Gaussian probability distributions centred on the foci and scaled according to sample size (each study's results are assumed to have a degree of spatial uncertainty).^13–16^ Then, an ALE map is created by computing the union of activation probabilities across all the included studies for each voxel.^13–16^ Finally, the ALE algorithm tests for true convergence of foci by testing against the null hypothesis of random spatial clustering between experiments.^13–16^ By using this approach iteratively, we could separately test for convergence of brain activity changes following psychotherapy, following antidepressants and during affect processing. This enabled us to perform a series of ALE conjunction and contrast analyses to identify distinct and shared loci of activation between the thresholded activation maps.

For our synthesis, we required two meta-analyses that met the following criteria:

- recently published, which we defined as having been published within the past 5 years;
- included both pre- and post-treatment neuroimaging measures;
- contained an adequate sample size for activation likelihood meta-analysis (approximately 17–20 studies are needed for ALE to be adequately powered to robustly detect an effect and ensure that results are not driven by single experiments^16^);
- employed relatively comparable in-scanner assessments on the majority of studies included in the meta-analysis (e.g. negative emotional valence contrasts).

We searched the literature for meta-analyses that met these criteria, and contacted the corresponding author of each meta-analysis, who shared their data for our analyses.

For both the antidepressant^11^ and psychotherapy^12^ meta-analyses, we ran an ALE meta-analysis on the following subsets of the original data.

- From the psychotherapy meta-analysis, which included 19 studies in the original meta-analysis, we included all pre- versus post-treatment studies reporting at least one coordinate (*K* = 17, where *K* is equal to the number of studies) (ALE analysis does not incorporate studies with no findings).
- The antidepressant meta-analysis originally included studies with healthy controls and those measuring the neural effects of acute antidepressant administration, with follow-up scans at multiple post-treatment time points or using multiple task contrasts. Because none of these additional studies were comparable with the data from the psychotherapy meta-analysis, we employed additional exclusion criteria. Specifically, from the antidepressant meta-analysis, which included 60 studies in the original meta-analysis, we included studies involving patients reporting the effects of a course of antidepressant treatment (i.e. not those reporting results following a single dose of antidepressant, nor those conducted on healthy controls). If a study reported more than one post-treatment time, we included only the contrast at the later date (e.g. 16 weeks rather than 8 weeks); if a study reported more than one contrast (e.g. sad > happy and sad > neutral activation), we included only the first contrast listed in the data file. The antidepressant meta-analysis included results from either within-participant analyses (pre- versus post-antidepressant treatment) or group × time interactions from mixed-design studies (*K* = 24). See supplementary materials available at https://doi.org/10.1192/bjp.2021.16 for details.

Our final sample included 619 patients with primary diagnoses of major depressive disorder (*n* = 332), post-traumatic stress disorder (*n* = 32), generalised anxiety disorder (*n* = 28), social anxiety disorder (*n* = 135), panic disorder (*n* = 59) or obsessive–compulsive disorder (*n* = 33); these diagnoses comprised the main and primary diagnosis of a given patient in the study. Patients were scanned (with functional magnetic resonance imaging, positron emission tomography or single-photon emission tomography) prior to and following either serotonin or noradrenaline reuptake inhibitor treatment (*n* = 343; 200 foci) or psychotherapy (majority CBT, but also mindfulness or other therapies)^12^ (*n* = 276; 120 foci). Task contrasts were largely comparable between the antidepressant and psychotherapy meta-analyses, with the vast majority reporting negative emotion valence contrasts (see supplementary Table 1 for task type and contrast, imaging and intervention type). A limitation of the comparability between the antidepressant and psychotherapy meta-analyses was the time from pre- to post-treatment scan. This varied substantially between studies (supplementary Table 1), ranging from 7 to 154 days for antidepressant medication and 56 to 182 days for psychotherapy; the median time between scans also differed significantly (56 and 84 days respectively; non-parametric Mann–Whitney U-test P < 0.001). This reflects inherent clinical differences between the two therapeutic approaches; psychotherapy is typically delivered on a weekly, fortnightly (or in some cases an even less-frequent) basis, whereas antidepressants are administered daily. We list all study details in supplementary Table 1, including the time between pre- and post-treatment scan.

We also extracted a subset of contrasts reported in a large database of affective task-based neuroimaging studies:^5^ whole-brain results for valenced affective stimuli contrasted with a neutral emotion baseline, producing 3869 foci from 216 experiments (*n* = 3587).

### Analysis

We tested for above-chance clustering following antidepressant medication or psychotherapy separately, using the random-effects model implemented by the ALE algorithm to acquire two family-wise error (FWE) cluster-corrected maps of convergence of changes following antidepressant treatment and psychological therapy. We thresholded maps at the recommended level for statistical significance in ALE meta-analysis,^14^ cluster-level FWE-corrected *P* < 0.05 (cluster-forming threshold at *P* < 0.001; 1000 threshold permutations). Then, we performed a contrast and conjunction analysis^17^ of psychotherapy versus antidepressant ALE maps (*P* < 0.05; 1000 permutations; minimum cluster size: 50 mm^3^). For this primary analysis, we report results from both corrected (*P* < 0.05 FWE cluster-corrected with *P* < 0.001 cluster-forming threshold) and uncorrected (*P* < 0.001 voxel-wise) maps.

For our follow-up analyses, after acquiring an FWE cluster-corrected map of convergence of activation during affective task-based neuroimaging studies (FWE-corrected threshold at *P* < 0.05; cluster-forming threshold at *P* < 0.001; 1000 threshold permutations), we performed two additional conjunction analyses comparing the corrected psychotherapy and antidepressant ALE maps each with the network of regions involved in affect processing^18^ (*P* < 0.05; 1000 permutations; minimum cluster size: 50 mm^3^).

## Results

**Fig. 1:**
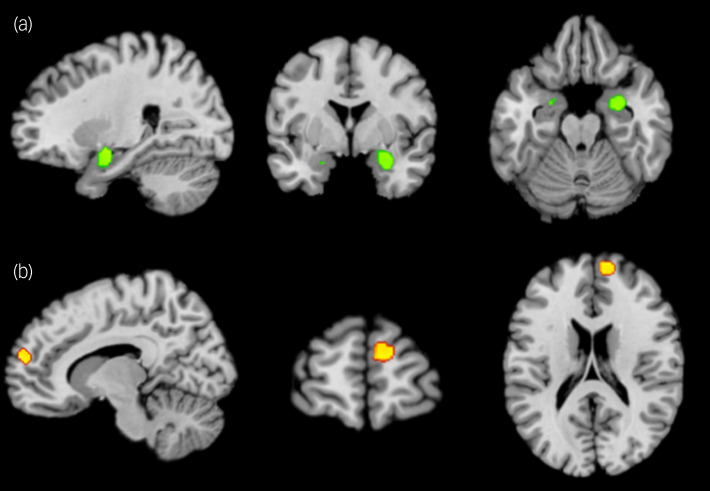
Neural changes following antidepressant treatment versus psychological therapy for affective disorders. (a) Preferential involvement of the bilateral amygdala and right medial globus pallidus in antidepressant treatment versus psychotherapy. (b) Preferential involvement of the medial prefrontal cortex in psychotherapy versus antidepressant treatment. No convergence of changes was found. All results thresholded at *P* < 0.05 family-wise error cluster-corrected (initial cluster-forming threshold *P* < 0.001). For display, *Z*-maps were overlaid onto a standard brain in MNI space (Colin27, a stereotaxic average of 27 single-subject anatomical scans, skull stripped) using Mango software (http://ric.uthscsa.edu/mango).

We found no overlapping neural changes following psychotherapy or antidepressants at either corrected (FWE cluster-corrected *P* < 0.05) or uncorrected (*P* < 0.001) thresholds. Following antidepressant treatment, there was preferential activation of the right amygdala extending to the right medial globus pallidus (Z = 3.09, *P* = 0.001; peak: 22, −6, −12; volume: 1704 mm^3^) and a smaller left amygdala cluster (Z = 1.66, *P* = 0.048; peak: −21, −1, −24; volume: 912 mm^3^) compared with psychotherapy. Following psychotherapy, there was preferential involvement of the medial prefrontal cortex (mPFC) (BA9) (Z = 2.33, *P* = 0.01; peak: 10, 62.7, 16.7; volume: 912 mm^3^) compared with antidepressant medication (Fig. 1; supplementary Table 2).

In our follow-up conjunction analyses, neural regions associated with affect processing overlapped with both the bilateral amygdala activation evoked by antidepressants (left: ALE = 0.02, volume 1848 mm^3^, peak −20, −6, −16; right: ALE = 0.024, volume 1696 mm^3^, peak 28, −4, 20) and the mPFC cluster evoked by psychotherapy (ALE = 0.014, volume 112 mm^3^, peak 8, 56, 18) (supplementary Fig. 1 and Table 1).

## Discussion

We demonstrate treatment-specific brain effects following antidepressant treatment versus psychotherapy, consistent with theories of different proximal mechanisms of action.^1^ Nevertheless, the effects of both interventions overlapped with a network involved in representing affective states.^5^ Psychotherapy is thought to target cognitive processes and ‘negative schemata’^19^ via prefrontal control over processing of affective information mediated by the limbic system.^2^ In the context of our findings, psychotherapy might alter attention and awareness of affective state through changes in mPFC function.^1^ In contrast, antidepressants might target the brain's affective or visceromotor state directly by altering limbic brain structures involved in generating a negative affective bias.^4^ One example of these brain structures is the amygdala, the central locus of our antidepressants results. Changes in the amygdala after antidepressant treatment (predominantly SSRIs) could have resulted from increased serotonin availability at the synapses, leading to amygdala inhibition.^1^

We show that the divergent effects of psychotherapy and antidepressant medication nevertheless overlap with the brain's affect network. This overlap might explain the enhanced efficacy of combined pharmacological and psychological treatment.^1^ The dorsomedial prefrontal cortex and the amygdala are both reliably engaged during affective (versus neutral) processing; nevertheless, they may participate in functionally dissociable processes. Previous work suggests that the dorsomedial prefrontal node is implicated in focusing conscious attention on feelings and the amygdala is implicated in driving changes in affective fluctuations.^20^ Psychotherapy and antidepressants also seem to differentially target these psychological processes, which may contribute to the observed findings and the enhanced efficiency of combined treatments – a possibility that could be explored in future research.

### Limitations

There is increasing recognition that the neurocognitive mechanisms of psychiatric disorders do not reflect traditional diagnostic categories.^21,22^ In line with this transdiagnostic approach, our meta-analysis included patients with various affective disorders, including major depressive disorder, panic disorder and generalised anxiety disorder. Our meta-analysis was powered to detect reasonably small convergent activation effects for psychotherapy and antidepressant treatments,^16^ but we did not have adequate statistical power to conduct subgroup analyses such as examining specific diagnostic categories, particular cognitive tasks or specific types of antidepressant. Therefore, it is possible that the convergence and divergence of psychotherapy and antidepressant effects may differ across particular primary diagnostic categories, with the caveat that even for these subgroups diagnostic comorbidity will be common in the participants.^21^ Similarly, our contrast and conjunction meta-analysis included a majority of negative-affect tasks and no positive-affect tasks, because we endeavoured to make our comparison analysis as comparable as possible (originally, only the antidepressant meta-analysis, and not the psychotherapy meta-analysis, reported positive-valence contrasts). Therefore, it is possible that different neural regions of convergence and divergence might arise when measuring changes in neural activation relating to positive affect.

Another important limitation of our meta-analysis is the difference in pre- to post-treatment scan times between psychotherapy and antidepressant studies. This methodological difference across the two therapeutic approaches could have affected our results, with antidepressants being delivered daily and assessed after less time, and psychotherapy being delivered less frequently and assessed after a longer time. These are inherent clinical differences between the two treatment modalities and it would be important for future work to address the question of what a comparable ‘dose’ between the two treatment approaches would be, and to measure pre- and post-treatment neural activation at this comparable interval.

To maximise our sample size, we included studies with a variety of different antidepressants and psychotherapy modalities. Previous work would suggest that some mechanisms of action are distinct between antidepressant types: the antidepressant meta-analysis included here^11^ found that only SSRIs evoked convergent amygdala changes, whereas studies employing serotonin–noradrenaline reuptake inhibitors (SNRIs) evoked other subcortical activation changes.^11^ Therefore, our result may have been driven in particular by SSRI-evoked changes, which could be tested by comparing future, larger meta-analyses of subtypes of antidepressant.

Note that our results demonstrate a divergence of neural effects of psychotherapy versus antidepressant medication, irrespective of whether or not a patient responded to the psychotherapy or antidepressant they received. A recent meta-analysis of neural biomarkers of treatment response for antidepressants found that heightened amygdala activation at baseline was associated with worse treatment response (along with heightened insula and striatal activation).^23^ Our meta-analysis was designed to test differences between antidepressant medication and psychotherapy, and consequently did not have the statistical power to separately examine neural changes in treatment responders versus non-responders for the two interventions. Therefore, our findings might be driven by patients whose symptoms reduced following the intervention, or might be indicative of general neural changes, irrespective of treatment response. If the latter, it is possible that the changes in amygdala activation caused by antidepressants that we report are insufficient to cause treatment response in patients with particularly high amygdala activation at baseline, despite a common reduction in activation. Future meta-analyses should focus on regions implicated specifically in treatment response. These mechanisms could eventually be compared with those neural changes following novel treatments (e.g. psychedelics; brain stimulation; ketamine) to determine whether their proximal mechanisms are overlapping or distinct from those involved in psychotherapy and antidepressant medication.

## Data Availability

The data that support the findings of this study are openly available on the Open Science Framework at https://osf.io/uh3w2/.

## Supplementary material

For supplementary material accompanying this paper visit https://doi.org/10.1192/bjp.2021.16. click here to view supplementary material
