# Supplementary material for: Neural effects of antidepressant medication and psychological treatments: a quantitative synthesis across three meta-analyses
Source: Br J Psychiatry. 2021 Oct;219(4):546–50. doi: 10.1192/bjp.2021.16 (PMC8481936; doi:10.1192/bjp.2021.16)
Supplement: Supplementary file 1 [file S0007125021000167sup001.docx]

Supplemental Materials

*Distinct neural effects of psychological therapy and antidepressant medication on the brain’s affect circuitry: a synthesis across three meta-analyses*

Camilla L Nord, PhD^1^, Lisa Feldman Barrett, PhD^2^, Kristen A. Lindquist, PhD^3^, Yina Ma, PhD^4^, Lindsey Marwood, PhD^5^, Ajay B Satpute, PhD^2^, Tim Dalgleish, PhD^1^

^1^ Medical Research Council Cognition and Brain Sciences Unit, University of Cambridge, 15 Chaucer Road, Cambridge, CB2 7EF

^2^ Department of Psychology, Northeastern University, Boston, MA, USA

^3^ Department of Psychology and Neuroscience, University of North Carolina, Chapel Hill, NC, USA

^4^ State Key Laboratory of Cognitive Neuroscience and Learning, IDG/McGovern Institute for Brain Research, Beijing Normal University, Beijing, China

^5^ Department of Psychological Medicine, Institute of Psychiatry, Psychology & Neuroscience, King’s College London, UK

*Inclusion criteria and procedure for synthesis of meta-analyses*

For both antidepressant medication (ADM)^1^ and psychological therapy (PT)^2^ meta-analyses, we ran an activation likelihood estimation (ALE) analysis on the following subsets of the original data:

From the PT meta-analysis, we included all pre- versus post-treatment studies reporting at least one coordinate from the original meta-analysis (K=17) (ALE analysis does not incorporate studies with no findings). To ensure this meta-analysis had sufficient and comparable statistical power for the contrast analysis with ADM, we did not exclude the four resting-state studies in the original meta-analysis. All other studies in both ADM and PT meta-analyses were task-based (see Table S1 for task type and contrast, imaging, and intervention type).

From the ADM meta-analysis, we included studies in patients reporting the effects of a course of antidepressant treatment (i.e., not those reporting results following a single dose of antidepressant administration, nor those conducted in healthy controls). If a study reported more than one post-treatment time, we included only the contrast at the later date (e.g. 16 weeks rather than 8 weeks); if a study reported more than one contrast (e.g., sad>happy and sad>neutral activation), we included only the first contrast listed in the data file. ADM meta-analyses included results from either within-subject analyses (pre- versus post-antidepressant treatment) or group-by-time interactions from mixed-design studies (K=24).

The contrasts were largely comparable between ADM and PT meta-analyses, with the vast majority reporting negative emotion valence contrasts (see Table S1 for specific contrasts). However, an important limitation of their comparability was four resting-state studies included in the psychological therapy meta-analysis. These four studies were included in the original meta-analysis for reasons of statistical power; without these four studies, the meta-analysis would be considered underpowered according to field-wide guidelines^3^. Therefore, we elected to include these studies with the caveat that it slightly diminishes the comparability of the samples.

After performing a standard ALE meta-analysis^4–6^ of PT and ADM data separately (to acquire a family-wise error (FWE) cluster-corrected map of convergence of changes following antidepressant treatment), we ran a conjunction analysis^47^ between the ADM and PT maps.

To compare PT and ADM effects with established ‘affect circuitry’ in the brain, we first extracted contrasts from a large database of affective task-based neuroimaging studies built for a previous meta-analysis^8^. The ‘affect circuitry’ studies we included represented a subset of the original database: we included only whole-brain (not region of interest [ROI]) results for valenced affect stimuli contrasted with a neutral emotion baseline. This produced 3867 foci from 216 experiments. After performing a standard ALE meta-analysis^4–6^ of the affect data (to acquire a FWE cluster-corrected map of convergence of affect-related activation), we ran a conjunction analysis with each equivalent map obtained from the ADM and PT meta-analyses above.

Note that the ALE algorithm compares the convergence of reported coordinates with those expected under random spatial association and tests for above-chance clustering using random-effects inference; foci are treated as three-dimensional Gaussian probability distributions centred on the coordinates and scaled according to sample size^4–6^. All three meta-analysis maps were thresholded at a cluster-level family-wise- error (FWE)-corrected threshold of *p*<0.05 (cluster-forming threshold at *p*<0.001; 1000 threshold permutations). The three conjunction analyses run were all set at a minimum volume of 50mm^3^ (*p*=0.05, 1000 *p*-value permutations), although we additionally verified the absence of any convergence between PT and ADM effects in an uncorrected *p*<0.001 conjunction analysis.

We list all study details in Table S1, including the time from pre- to post-treatment scan, which differed between ADM and PT (median 56 and 84 days, respectively; non-parametric Mann-Whitney U test *p*<0.001).

Our FWE-corrected results for all conjunction and contrast meta-analyses can be found in **Table S2** (*Convergence and divergence of neural changes following antidepressant medication (ADM) and psychological therapy (PT) for affective disorders*).

**Table S1. Details of studies included in PT and ADM contrast (N=613)**

| First author | Year | Diagnosis | Intervention | N patients | Task | Contrast | Imaging | Time |
| --- | --- | --- | --- | --- | --- | --- | --- | --- |
| Anand^9^ | 2007 | MDD | SSRI | 12 | emotion | negative emotion>fixation | fMRI | 42 |
| Arnone^10^ | 2012 | social phobia | SSRI | 30 | emotion | sad>neutral | fMRI | 56 |
| Aupperle^11^ | 2013 | PTSD | CBT (CTT) | 14 | emotion | negative>positive | fMRI | 28 |
| Benedetti^12^ | 2009 | MDD | SNRI | 8 | emotion | negative>positive | fMRI | 21 |
| Cornelius^13^ | 2010 | MDD | SSRI | 6 | emotion | fear>shape | fMRI | 56 |
| Davidson^14^ | 2003 | MDD | SNRI | 12 | emotion | negative>neutral | fMRI | 56 |
| Fales^15^ | 2009 | MDD | SSRI | 23 | emotion | fear>neutral | fMRI | 28 |
| Felmingham^16^ | 2007 | PTSD | CBT | 8 | emotion | fearful>neutral | fMRI | 56 |
| Frodl^17^ | 2011 | MDD | SNRI | 11 | emotion | sad>shapes | fMRI | 7 |
| Fu^18^ | 2004 | MDD | SSRI | 13 | emotion | sad>fixation | fMRI | 49 |
| Furmark^19^ | 2002 | social phobia | CBT | 6 | emotion | anxiogenic public speaking | PET | 14 |
| Godlewska^20^ | 2012 | MDD | SSRI | 42 | emotion | fear>happy | fMRI | 84 |
| Goldapple^21^ | 2004 | MDD | CBT | 14 | resting | resting | PET | 56 |
| Goldin^22^ | 2010 | SAD | mindfulness | 14 | emotion | negative self-belief>fixation | fMRI | 56 |
| Goldin^23^ | 2012 | SAD | CBT | 24 | emotion | negative self-referential>self | fMRI | 84 |
| Hoehn-Saric^24^ | 2004 | GAD | SSRI | 6 | emotion | worry>neutral | fMRI | 56 |
| Holzel^25^ | 2013 | GAD | mindfulness | 15 | emotion | angry>neutral | fMRI | 56 |
| Kalin^26^ | 1997 | MDD | SNRI | 2 | emotion | negative>neutral | fMRI | 84 |
| Keedwell^27^ | 2009 | MDD | variety | 12 | emotion | sad>fixation | fMRI | 28 |
| Kircher^28^ | 2013 | PD | CBT | 42 | emotion | fear-conditioned> non-conditioned | fMRI | 154 |
| Klumpp^29^ | 2013 | SAD | CBT | 14 | emotion | fearful>happy | fMRI | 42 |
| Lindauer^30^ | 2008 | PTSD | BEP | 10 | emotion | symptom provocation | SPECT | 56 |
| Lopez-Sola^31^ | 2010 | MDD | SNRI | 13 | pain | painful>nonpainful | fMRI | 56 |
| Mansson^32^ | 2013 | SAD | CBT (iCBT) | 13 | emotion | disgust>neutral | fMRI | 56 |
| Mansson^33^ | 2013 | SAD | ABM | 13 | emotion | disgust>neutral | fMRI | 84 |
| Maslowsky^34^ | 2010 | GAD | SSRI | 7 | emotion | angry>fixation | fMRI | 182 |
| Phan^35^ | 2012 | social phobia | SSRI | 21 | emotion | fear>happy | fMRI | 63 |
| Prasko^36^ | 2004 | PD | CBT | 6 | resting | resting | PET | 42 |
| Robertson^37^ | 2007 | MDD | SNRI | 10 | emotion | sad>neutral | fMRI | 182 |
| Rosenblau^38^ | 2012 | MDD | SSRI | 12 | emotion | negative>positive | fMRI | 56 |
| Ruhe^39^ | 2012 | MDD | SSRI | 16 | emotion | fear>scrambled faces | fMRI | 56 |
| Sakai^40^ | 2006 | PD | CBT | 11 | resting | resting | PET | 56 |
| Samson^41^ | 2011 | MDD | SSRI/SNRI | 10 | emotion | sad>baseline | fMRI | 84 |
| Sankar^42^ | 2015 | MDD | CBT | 16 | emotion | negative attitudes>neutral | fMRI | 112 |
| Schaefer^43^ | 2006 | MDD | SNRI | 9 | social | social interaction>other | fMRI | 63 |
| Stoy^44^ | 2012 | MDD | SSRI | 15 | reward | loss>neutral | fMRI | 63 |
| Tao^45^ | 2012 | adolescent MDD | SSRI | 19 | emotion | fear>neutral | fMRI | 84 |
| Victor^46^ | 2010 | MDD | SSRI | 10 | emotion | sad>neutral | fMRI | 182 |
| Wang^47^ | 2012 | MDD | SSRI | 18 | emotion | negative>neutral | fMRI | 112 |
| Yamanishi^48^ | 2009 | OCD | BT | 33 | resting | resting | SPECT | 84 |
| Yoshimura^49^ | 2014 | MDD | CBT | 23 | emotion | negative self-referential>verbal | fMRI | 84 |

*MDD=Major depressive disorder, GAD=generalized anxiety disorder, PD=panic disorder, PTSD=post-traumatic stress disorder, OCD=Obsessive-compulsive disorder, SAD=social anxiety disorder; fMRI=functional magnetic resonance imaging; PET=positron emission tomography; SPECT=single photon emission tomography; SSRI=selective serotonin reuptake inhibitor; SNRI=selective noradrenaline reuptake inhibitor; CBT=cognitive behavioural therapy; iCBT=internet-based CBT; CTT=cognitive trauma therapy; BT=behavioural therapy; ABM=affective bias modification; BEP=brief eclectic psychotherapy; neg=negative. Time = time from pre- to post-treatment scan reported in the paper, rounded to the nearest day.*

**Table S2. Convergence and divergence of neural changes following antidepressant medication (ADM) and psychological therapy (PT) for affective disorders.**

*Convergence of changes following ADM and PT (corrected and uncorrected results)*

| Region | MNI coordinates | Volume (mm^3^) |  |  |
| --- | --- | --- | --- | --- |

*x y z*

| No regions |  |  |  |  |  |  |
| --- | --- | --- | --- | --- | --- | --- |

*Contrast: Changes following ADM minus PT (cluster-corrected)*

| Region | MNI coordinates | Volume (mm^3^) | *Z* | *P* |
| --- | --- | --- | --- | --- |

*x y z*

| R Med. GP/amygdala | 22 | -6 | -12 | 1704 | 3.09 | 0.001 |
| --- | --- | --- | --- | --- | --- | --- |
| R Amygdala | 30 | -6 | -14 |  | 2.37 | 0.009 |
| R Amygdala | 25.1 | -8.3 | -20.3 |  | 2.33 | 0.01 |
| R Amygdala | 29.5 | -5.5 | -19 |  | 2.29 | 0.011 |
| R Amygdala | 28 | -6 | -26 |  | 2.26 | 0.012 |
| R Amygdala | 31.3 | -1.3 | -16.7 |  | 2.23 | 0.013 |
| R Amygdala | 26 | 2 | -24 |  | 2.12 | 0.017 |
| L Amygdala | -21 | -1 | -24 | 912 | 1.66 | 0.048 |

*Contrast: Changes following PT minus ADM (cluster-corrected)*

| Region | MNI coordinates | Volume (mm^3^) | *Z* | *P* |
| --- | --- | --- | --- | --- |

*x y z*

| Med.  PFC | 10 | 62.7 | 16.7 | 912 | 2.33 | 0.010 |
| --- | --- | --- | --- | --- | --- | --- |
|  | 12.3 | 57.4 | 15.1 |  | 2.20 | 0.014 |
|  | 12 | 56 | 13 |  | 2.20 | 0.014 |
|  | 10 | 60 | 22 |  | 2.14 | 0.016 |

*Convergence of changes following ADM and affect network (cluster-corrected)*

| Region | MNI coordinates | Volume (mm^3^) | *ALE* |  |
| --- | --- | --- | --- | --- |

*x y z*

| L Amygdala | -20 | -6 | -16 | 1848 | 0.020 |  |
| --- | --- | --- | --- | --- | --- | --- |
| R Amygdala | 28 | -4 | -20 | 1696 | 0.024 |  |

*Convergence of changes following PT and affect network (cluster-corrected)*

| Region | MNI coordinates | Volume (mm^3^) | *ALE* |  |
| --- | --- | --- | --- | --- |

*x y z*

| Med. PFC | 8 | 56 | 18 | 112 | 0.014 |  |
| --- | --- | --- | --- | --- | --- | --- |

Family-wise error (FWE) cluster-corrected results for peak coordinates of each cluster. Initial ALE maps thresholded at *p*<0.05 FWE cluster-corrected (cluster-forming threshold *p*<0.001); subsequent conjunction/contrast analysis thresholded at *p*<0.05; 1000 *p*-value permutations; minimum volume for conjunction/contrast: 50mm^3^. MNI=Montreal Neurological Institute; L=left; R=right; Med.=medial; GP=globus pallidus; PFC=prefrontal cortex; PT=psychological therapy; ADM=antidepressant medication.

**References**

1. Ma Y. Neuropsychological mechanism underlying antidepressant effect: a systematic meta-analysis. *Molecular psychiatry*. 2015;20(3):311-319.

2. Marwood L, Wise T, Perkins AM, Cleare AJ. Meta-analyses of the neural mechanisms and predictors of response to psychotherapy in depression and anxiety. *Neuroscience & Biobehavioral Reviews*. 2018;95:61-72.

3. Müller VI, Cieslik EC, Laird AR, et al. Ten simple rules for neuroimaging meta-analysis. *Neuroscience & Biobehavioral Reviews*. 2018;84:151-161.

4. Eickhoff SB, Laird AR, Grefkes C, Wang LE, Zilles K, Fox PT. Coordinate‐based activation likelihood estimation meta‐analysis of neuroimaging data: A random‐effects approach based on empirical estimates of spatial uncertainty. *Human brain mapping*. 2009;30(9):2907-2926.

5. Eickhoff SB, Bzdok D, Laird AR, Kurth F, Fox PT. Activation likelihood estimation meta-analysis revisited. *Neuroimage*. 2012;59(3):2349-2361.

6. Turkeltaub PE, Eickhoff SB, Laird AR, Fox M, Wiener M, Fox P. Minimizing within‐experiment and within‐group effects in activation likelihood estimation meta‐analyses. *Human brain mapping*. 2012;33(1):1-13.

7. Eickhoff SB, Bzdok D, Laird AR, et al. Co-activation patterns distinguish cortical modules, their connectivity and functional differentiation. *Neuroimage*. 2011;57(3):938-949.

8. Lindquist KA, Satpute AB, Wager TD, Weber J, Barrett LF. The brain basis of positive and negative affect: evidence from a meta-analysis of the human neuroimaging literature. *Cerebral cortex*. 2016;26(5):1910-1922.

9. Anand A, Li Y, Wang Y, Gardner K, Lowe MJ. Reciprocal effects of antidepressant treatment on activity and connectivity of the mood regulating circuit: an FMRI study. *The Journal of neuropsychiatry and clinical neurosciences*. 2007;19(3):274-282.

10. Arnone D, McKie S, Elliott R, et al. Increased amygdala responses to sad but not fearful faces in major depression: relation to mood state and pharmacological treatment. *American Journal of Psychiatry*. 2012;169(8):841-850.

11. Aupperle RL, Allard CB, Simmons AN, et al. Neural responses during emotional processing before and after cognitive trauma therapy for battered women. *Psychiatry Research: Neuroimaging*. 2013;214(1):48-55. doi:10.1016/j.pscychresns.2013.05.001

12. Benedetti F, Bernasconi A, Radaelli D, et al. Changes in medial prefrontal cortex neural responses parallel successful antidepressant combination of venlafaxine and light therapy. *The International Journal of Neuropsychopharmacology*. 2008;11:97.

13. Cornelius JR, Aizenstein HJ, Hariri AR. Amygdala reactivity is inversely related to level of cannabis use in individuals with comorbid cannabis dependence and major depression. *Addictive behaviors*. 2010;35(6):644-646.

14. Davidson RJ, Irwin W, Anderle MJ, Kalin NH. The neural substrates of affective processing in depressed patients treated with venlafaxine. *American journal of Psychiatry*. 2003;160(1):64-75.

15. Fales CL, Barch DM, Rundle MM, et al. Antidepressant treatment normalizes hypoactivity in dorsolateral prefrontal cortex during emotional interference processing in major depression. *Journal of affective disorders*. 2009;112(1):206-211.

16. Felmingham K, Kemp A, Williams L, et al. Changes in Anterior Cingulate and Amygdala After Cognitive Behavior Therapy of Posttraumatic Stress Disorder. *Psychol Sci*. 2007;18(2):127-129. doi:10.1111/j.1467-9280.2007.01860.x

17. Frodl T, Scheuerecker J, Schoepf V, et al. Different effects of mirtazapine and venlafaxine on brain activation: an open randomized controlled fMRI study. *The Journal of clinical psychiatry*. 2010;72(4):448-457.

18. Fu CH, Williams SC, Cleare AJ, et al. Attenuation of the neural response to sad faces in major depressionby antidepressant treatment: a prospective, event-related functional magnetic resonance imagingstudy. *Archives of general psychiatry*. 2004;61(9):877-889.

19. Furmark T, Tillfors M, Marteinsdottir I, et al. Common Changes in Cerebral Blood Flow in Patients With Social Phobia Treated With Citalopram or Cognitive-Behavioral Therapy. *Archives of General Psychiatry*. 2002;59(5):425-433. doi:10.1001/archpsyc.59.5.425

20. Godlewska B, Norbury R, Selvaraj S, Cowen P, Harmer C. Short-term SSRI treatment normalises amygdala hyperactivity in depressed patients. *Psychological medicine*. 2012;42(12):2609-2617.

21. Goldapple K, Segal Z, Garson C, et al. Modulation of cortical-limbic pathways in major depression: treatment-specific effects of cognitive behavior therapy. *Archives of general psychiatry*. 2004;61(1):34-41.

22. Goldin PR, Gross JJ. Effects of mindfulness-based stress reduction (MBSR) on emotion regulation in social anxiety disorder. *Emotion*. 2010;10(1):83-91. doi:10.1037/a0018441

23. Goldin PR, Ziv M, Jazaieri H, et al. Cognitive reappraisal self-efficacy mediates the effects of individual cognitive-behavioral therapy for social anxiety disorder. *J Consult Clin Psychol*. 2012;80(6):1034-1040. doi:10.1037/a0028555

24. Hoehn-Saric R, Schlund MW, Wong SH. Effects of citalopram on worry and brain activation in patients with generalized anxiety disorder. *Psychiatry Research: Neuroimaging*. 2004;131(1):11-21.

25. Hölzel BK, Hoge EA, Greve DN, et al. Neural mechanisms of symptom improvements in generalized anxiety disorder following mindfulness training. *NeuroImage: Clinical*. 2013;2:448-458. doi:10.1016/j.nicl.2013.03.011

26. Kalin NH, Davidson RJ, Irwin W, et al. Functional magnetic resonance imaging studies of emotional processing in normal and depressed patients: effects of venlafaxine. *The Journal of Clinical Psychiatry*. Published online 1997.

27. Keedwell P, Drapier D, Surguladze S, Giampietro V, Brammer M, Phillips M. Neural markers of symptomatic improvement during antidepressant therapy in severe depression: subgenual cingulate and visual cortical responses to sad, but not happy, facial stimuli are correlated with changes in symptom score. *Journal of Psychopharmacology*. 2009;23(7):775-788.

28. Kircher T, Arolt V, Jansen A, et al. Effect of Cognitive-Behavioral Therapy on Neural Correlates of Fear Conditioning in Panic Disorder. *Biological Psychiatry*. 2013;73(1):93-101. doi:10.1016/j.biopsych.2012.07.026

29. Klumpp H, Fitzgerald DA, Phan KL. Neural predictors and mechanisms of cognitive behavioral therapy on threat processing in social anxiety disorder. *Progress in Neuro-Psychopharmacology and Biological Psychiatry*. 2013;45:83-91.

30. Lindauer R, Booij J, Habraken J, et al. Effects of psychotherapy on regional cerebral blood flow during trauma imagery in patients with post-traumatic stress disorder: A randomized clinical trial. *Psychological medicine*. 2008;38:543-554. doi:10.1017/S0033291707001432

31. López-Solà M, Pujol J, Hernández-Ribas R, et al. Effects of duloxetine treatment on brain response to painful stimulation in major depressive disorder. *Neuropsychopharmacology*. 2010;35(11):2305-2317.

32. Månsson KNT, Carlbring P, Frick A, et al. Altered neural correlates of affective processing after internet-delivered cognitive behavior therapy for social anxiety disorder. *Psychiatry Research: Neuroimaging*. 2013;214(3):229-237. doi:10.1016/j.pscychresns.2013.08.012

33. Månsson K, al et. *Amygdala Changes after Cognitive Behavior Therapy and Attention Bias Modification via the Internet – an FMRI-Study*.; 2013.

34. Maslowsky J, Mogg K, Bradley BP, et al. A preliminary investigation of neural correlates of treatment in adolescents with generalized anxiety disorder. *Journal of child and adolescent psychopharmacology*. 2010;20(2):105-111.

35. Phan KL, Coccaro EF, Angstadt M, et al. Corticolimbic brain reactivity to social signals of threat before and after sertraline treatment in generalized social phobia. *Biological Psychiatry*. 2013;73(4):329-336.

36. Prasko J, Horacek J, Záleský R, et al. The change of regional brain metabolism (18FDG PET) in panic disorder during the treatment with cognitive behavioral therapy or antidepressants. *Neuro endocrinology letters*. 2004;25:340-348.

37. Robertson B, Wang L, Diaz MT, et al. Effect of bupropion extended release on negative emotion processing in major depressive disorder: a pilot functional magnetic resonance imaging study. *The Journal of clinical psychiatry*. Published online 2007.

38. Rosenblau G, Sterzer P, Stoy M, et al. Functional neuroanatomy of emotion processing in major depressive disorder is altered after successful antidepressant therapy. *Journal of psychopharmacology*. 2012;26(11):1424-1433.

39. Ruhé HG, Mason NS, Schene AH. Mood is indirectly related to serotonin, norepinephrine and dopamine levels in humans: a meta-analysis of monoamine depletion studies. *Molecular psychiatry*. 2007;12(4):331.

40. Sakai Y, Kumano H, Nishikawa M, et al. Changes in cerebral glucose utilization in patients with panic disorder treated with cognitive–behavioral therapy. *NeuroImage*. 2006;33(1):218-226. doi:10.1016/j.neuroimage.2006.06.017

41. Samson AC, Meisenzahl E, Scheuerecker J, et al. Brain activation predicts treatment improvement in patients with major depressive disorder. *Journal of psychiatric research*. 2011;45(9):1214-1222.

42. Sankar A, Scott J, Paszkiewicz A, Giampietro VP, Steiner H, Fu CHY. Neural effects of cognitive–behavioural therapy on dysfunctional attitudes in depression. *Psychological Medicine*. 2015;45(7):1425-1433. doi:10.1017/S0033291714002529

43. Schaefer HS, Putnam KM, Benca RM, Davidson RJ. Event-related functional magnetic resonance imaging measures of neural activity to positive social stimuli in pre-and post-treatment depression. *Biological psychiatry*. 2006;60(9):974-986.

44. Stoy M, Schlagenhauf F, Sterzer P, et al. Hyporeactivity of ventral striatum towards incentive stimuli in unmedicated depressed patients normalizes after treatment with escitalopram. *Journal of psychopharmacology*. 2012;26(5):677-688.

45. Tao R, Calley CS, Hart J, et al. Brain activity in adolescent major depressive disorder before and after fluoxetine treatment. *American Journal of Psychiatry*. 2012;169(4):381-388.

46. Victor TA, Furey ML, Fromm SJ, Öhman A, Drevets WC. Relationship between amygdala responses to masked faces and mood state and treatment in major depressive disorder. *Archives of general psychiatry*. 2010;67(11):1128-1138.

47. Wang Y, Xu C, Cao X, et al. Effects of an antidepressant on neural correlates of emotional processing in patients with major depression. *Neuroscience letters*. 2012;527(1):55-59.

48. Yamanishi T, Nakaaki S, Omori IM, et al. Changes after behavior therapy among responsive and nonresponsive patients with obsessive-compulsive disorder. *Psychiatry Research: Neuroimaging*. 2009;172(3):242-250. doi:10.1016/j.pscychresns.2008.07.004

49. Yoshimura S, Okamoto Y, Onoda K, et al. Cognitive behavioral therapy for depression changes medial prefrontal and ventral anterior cingulate cortex activity associated with self-referential processing. *Social Cognitive and Affective Neuroscience*. 2013;9(4):487-493. doi:10.1093/scan/nst009
